# Supplementary material for: Efficient isolation of rare B cells using next-generation antigen barcoding
Source: Front Cell Infect Microbiol. 2023 Mar 10;12:962945. doi: 10.3389/fcimb.2022.962945 (PMC10036767; doi:10.3389/fcimb.2022.962945)

Supplementary Material

1 Supplementary Tables

Supplementary Table 1. Barcoded oligonucleotides for AgBC construction.

name	barcode
AgBC1	TAGGCGTCGATGCCG
AgBC2	ACCGATACTCGACAT
AgBC3	TGTCGACATCGCCCC
AgBC4	TCCCCGGCAGTAGAA
AgBC5	CGGTAGGATCCACGC
AgBC6	GTCACAAGTGACATC
AgBC7	GATGCGCACGAACCG
AgBC8	CTAAAGGTTTATATC
AgBC9	GCTAGTTCGGCTTAT
AgBC10	TTACCCTAGACAAGC

Supplementary Table 2. Preparation of baits based on molecular weight.

	BG505 SOSIP	eOD-GT8.1
bait concentration	200 nM	200 nM
molar ratio of bait:SAV	2:1	4:1
bait molecular weight	225 kD	22 kD

Supplementary Table 3. Cell staining master mix (by species).

specificity	mouse	human
CD3e	-	APC-Cy7
CD4	APC-Cy7	APC-Cy7
CD8a	APC-Cy7	APC-Cy7
Ly6C	APC-Cy7	-
CD11c	APC-Cy7	-
CD14	-	APC-Cy7
F4/80	APC-Cy7	-
CD19	PE	PerCP-Cy5.5
IgD	PerCP-Cy5.5	-
IgM	BV786	PE
IgG	-	BV786

2 Supplementary Figures

Supplementary Figure 1. Genes distinguishing naïve, memory and atypical B cell subsets. Plot was created in scanpy using `scanpy.pl.dotplot()`.

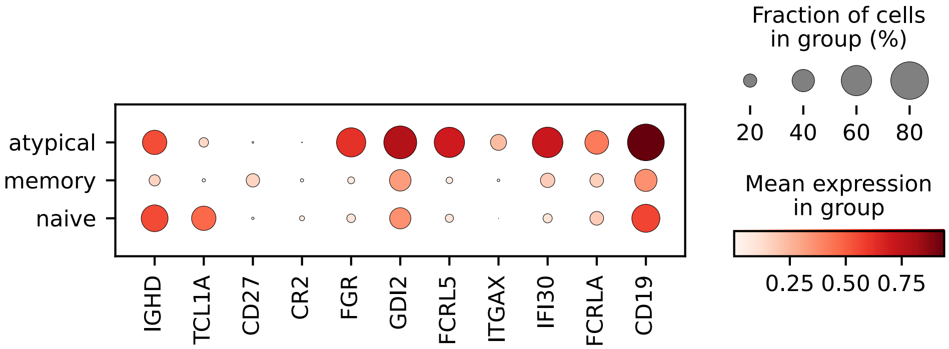

Supplement: Supplementary file 1 [file DataSheet_1.pdf]
